# Grazing Intensity Alters Leaf and Spike Photosynthesis, Transpiration, and Related Parameters of Three Grass Species on an Alpine Steppe in the Qilian Mountains

**DOI:** 10.3390/plants10020294

**Published:** 2021-02-04

**Authors:** Jin Li, Fujiang Hou, Jizhou Ren

**Affiliations:** State Key Laboratory of Grassland Agro-Ecosystems, Key Laboratory of Grassland Livestock Industry Innovation, Ministry of Agriculture and Rural Affairs, College of Pastoral Agriculture Science and Technology, Lanzhou University, Lanzhou 730000, China; li_jin@lzu.edu.cn (J.L.); renjz@vip.sina.com (J.R.)

**Keywords:** grazing intensity, alpine steppe, leaf, spike, photosynthesis parameters, photosynthetic pigments

## Abstract

The effect of grazing on leaf photosynthesis has been extensively studied. However, the influence of grazing on photosynthesis in other green tissues, especially spike, has remained poorly understood. This study investigated the impact of different grazing intensities (light grazing (LG), medium grazing (MG), and heavy grazing (HG)) on leaf and spike photosynthesis parameters and photosynthetic pigments of three grass species (*Stipa purpurea*, *Achnatherum inebrians*, and *Leymus secalinus*) on an alpine steppe in the Qilian Mountains. Grazing promoted leaf photosynthesis rate in *S. purpurea* and *L. secalinus* but reduced it in *A. inebrians*. Conversely, spike photosynthesis rate decreased in *S. purpurea* and *L. secalinus* under intense grazing, while there was no significant difference in spike photosynthesis rate in *A. inebrians*. The leaf and spike net photosynthetic rate (Pn) and transpiration rate (Tr) in *S. purpurea* were the greatest among the three species, while their organ temperatures were the lowest. On the other hand, grazing stimulated leaf chlorophyll biosynthesis in *S. purpurea* and *L. secalinus* but accelerated leaf chlorophyll degradation in *A. inebrians*. Furthermore, spike chlorophyll biosynthesis was inhibited in the three species under grazing, and only *L. secalinus* had the ability to recover from the impairment. Grazing had a positive effect on leaf photosynthesis parameters of *S. purpurea* and *L. secalinus* but a negative effect on those of *A. inebrians*. However, spike photosynthesis parameters were negatively influenced by grazing. Among the three species investigated, *S. purpurea* displayed the greatest ability for leaf and spike photosynthesis to withstand and acclimate to grazing stress. This study suggests that moderate grazing enhanced leaf photosynthetic capacity of *S. purpurea* and *L. secalinus* but reduced it in *A. inebrians*. However, spike photosynthetic capacity of three grass species decreased in response to grazing intensities.

## 1. Introduction

Rangelands cover more than 50% of the global terrestrial surface, and grazing is one of the most important utilizations of rangelands [1,2]. Photosynthesis, the most fundamental and intricate physiological process in all green plants and the primary source of energy for all life, contributes substantially to plant growth and development [3,4]. Owing to the combined effects of foraging, trampling, and excretion by livestock, grazing exerts a strong effect on the morphological and physiological characteristics of plants, the physical and chemical properties of the soil, and various abiotic and biotic factors, thus influencing the photosynthesis of rangelands [5,6,7]. Previous studies have shown that the effects of grazing on photosynthesis vary with grazing intensity and frequency [6,8]; that is, grazing contributes to either enhancing or reducing photosynthesis [9,10].

Leaves have long been considered the major photosynthetic organs in plants [11]. However, in addition to leaves, nonfoliar green organs containing chlorophyll, which exist on nearly all vegetative and reproductive structures in higher plants, can also be photosynthetically active and can perform photosynthetic CO_2_ assimilation [12,13]. To date, research on photosynthesis of nonfoliar organs has been performed mostly in crops species because photosynthesis of nonfoliar organs substantially contributes to crop growth and productivity, and more than 90% of crop biomass is derived from photosynthesis products assimilated by chlorenchyma tissue [12,13].

Among nonfoliar organs, spikes are optimized for active photosynthesis because their location is suitable for intercepting and capturing light and CO_2_. Spikes are composed of glumes, paleae, lemmata, awns, and rachides, which contain chlorophyll [14,15]. Furthermore, ear photosynthesis, which contributes 45–65% of the total grain filling for cereal species, is thought to make significant contributions in terms of the source of photoassimilates [16]. Owing to their delayed senescence, certain reproductive organs may play the same essential role as leaf photosynthesis but at a later stage of plant growth [14,17]. Moreover, reproductive organs often seem to be more resistant than leaves to certain abiotic stresses, which may suggest important adaptive characteristics [14,18]. Hence, because spikes are reproductive organs, research on spike photosynthesis has mainly focused on the different spike structures among cereals, such as wheat, barley, oats, and rice, under drought, high temperature, and heat stress [19,20,21,22]. However, little is known about how grazing impacts spike photosynthesis in graminoids such as *Stipa purpurea*, *Achnatherum inebrians*, and *Leymus secalinus*, which bear spikes as cereal crops.

Grassland degradation induced by overgrazing has become a severe problem on the northern slopes of the Qilian Mountains. The Qilian Mountains, located on the northeastern margin of the Qinghai–Tibetan Plateau in northwestern China, are of extraordinary importance as a water source for the lower reaches of the inland Heihe, Shiyang, and Shule rivers of the Hexi Corridor; these mountains have a crucial influence on ecosystem and water resource use within the Hexi Oasis [23,24,25]. Furthermore, by virtue of considerable glacier and water resource reserves, the Qilian Mountains are also vital ecological shelters in northwestern China [26,27]. As an important region for eco-environmental research, the northern slopes of the Qilian Mountains are not only a natural barrier between the Gobi Desert and the Qinghai–Tibetan Plateau but also a major base for animal husbandry in China [27]. Although transhumance pastoral systems are still in use in this area, which has a long history of livestock grazing, overgrazing has always been common because grazing pressure depends on the livestock number rather than the carrying capacity of grasslands [28]. The mean stocking rate in this area (0.94 sheep ha^−1^) was once 59% greater than the calculated stocking rate (0.59 sheep ha^−1^) for sustainable grazing [29].

In the study area, both *S. purpurea* and *L. secalinus* are dominant in the upper canopy layer of lightly and moderately grazed communities, while *A. inebrians* is dominant in the upper canopy layer of lightly, moderately, and heavily grazed communities. However, little is known about how leaf and spike photosynthesis parameters of these three grass species respond to grazing and about their differences in avoidance and tolerance strategies under the different grazing intensities. Therefore, we analyzed how different levels of grazing intensity influenced (i) photosynthesis parameters (net photosynthetic rate (Pn), transpiration rate (Tr), water use efficiency (WUE), leaf temperature (T_l_), and spike temperature (T_s_)); and (ii) photosynthetic pigments (chlorophyll a (Chl *a*), chlorophyll b (Chl *b*), total chlorophyll (TC), carotenoid (Car), chlorophyll a/chlorophyll b ratio (Chl *a*/*b*), and total chlorophyll/total carotenoid ratio (TC/Car). Specifically, the objectives of this study were as follows: (i) to examine the photosynthetic response and adaption of the three dominant grass species at different grazing intensities; (ii) to clarify differences in species’ strategies in response to grazing intensities; (iii) to explore the difference in the photosynthesis parameters between leaves and spikes of the three grass species under the different grazing intensities. We hypothesized (i) that owing to the compensatory photosynthesis, moderate grazing can enhance plant photosynthetic capacity; (ii) grazing intensity may have a different impact on photosynthetic capacity of *A. inebrians* due to its toxicity, which is a species strategy different from the other two species; (iii) due to the distinct spatial location, the photosynthetic capacity of leaf and spike may respond differently to the same grazing intensity.

## 2. Results

### 2.1. Environmental Parameters

**Figure 1:**
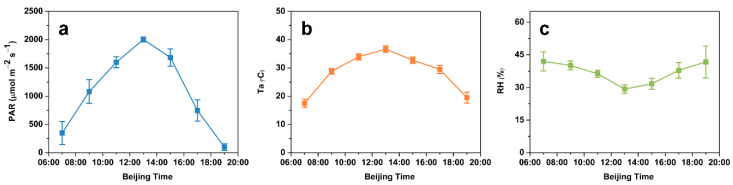
Diurnal time course of the photosynthetically active radiation (PAR) (**a**), air temperature (Ta) (**b**), and air relative humidity (RH) (**c**). The data are presented as mean ± standard error (SE) of three independent experiments.

Conditions were clear and sunny on the days when photosynthesis was measured. The changes in photosynthetically active radiation (PAR), air temperature (Ta), and air relative humidity (RH) were reasonably similar each day (Figure 1). The curve of the diurnal variation of the PAR had a notable single peak on a sunny day. The daily greatest PAR value was achieved at 13:00, peaking at 2002.96 µmol m^−2^ s^−1^ and decreasing thereafter. There was similar trend in the Ta diurnal pattern, with a peak value of 36.65 °C at 13:00 and gradually declining in the afternoon. Conversely, different from that which occurred for the PAR and Ta, the diurnal change in the RH presented a slightly valley at 13:00 and varied between 29.29 and 41.99%.

### 2.2. Photosynthesis Parameters

#### 2.2.1. Net Photosynthetic Rate

**Figure 2:**
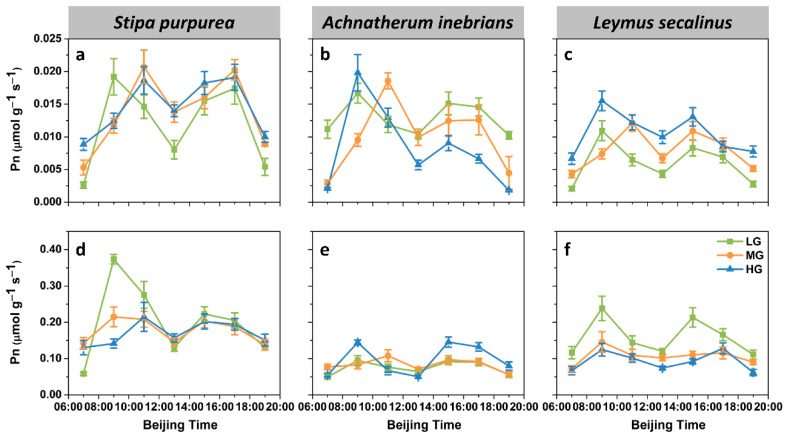
Diurnal dynamics of the net photosynthetic rate (Pn) of the leaves and spikes of *S. purpurea* (**a**,**d**), *A. inebrians* (**b**,**e**) and *L. secalinus* (**c**,**f**) in response to grazing intensity (LG, light grazing; MG, medium grazing; HG, heavy grazing). The data are presented as mean ± standard error (SE) of three independent experiments.

Regardless grazing intensity, leaf Pn of the three species all showed a bimodal pattern, with peak values in the morning (09:00–11:00) and afternoon (15:00–17:00) and an apparent midday depression at 13:00 (Figure 2a–c). The trends of the Pn patterns of the spikes were the same as those of the leaves for each species (Figure 2d–f). Among the three species, *S. purpurea* had the highest daily mean Pn values for both leaf and spike.

**Figure 3:**
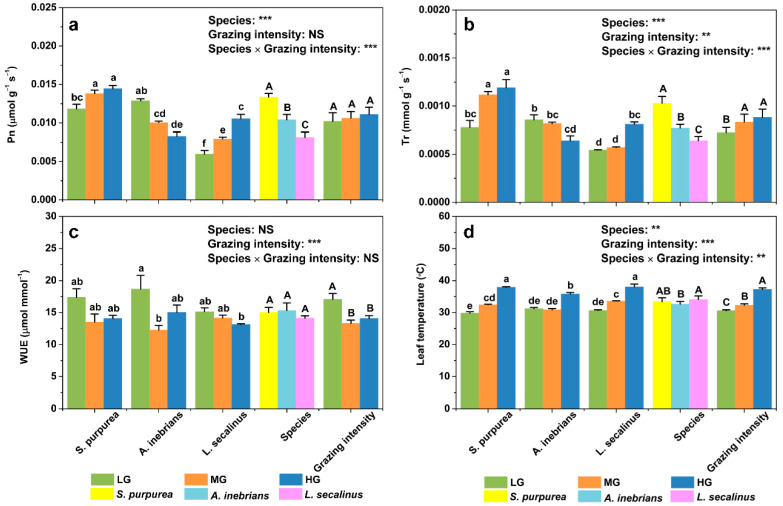
Mean values of photosynthesis parameters including the net photosynthetic rate (Pn) (**a**), transpiration rate (Tr) (**b**), water use efficiency (WUE) (**c**), and leaf temperature (T_l_) (**d**) of the leaves of *S. purpurea*, *A. inebrians* and *L. secalinus* in response to grazing intensity (LG, light grazing; MG, medium grazing; HG, heavy grazing). Note: *S. purpurea*, *Stipa purpurea*; *A. inebrians*, *Achnatherum inebrians*; *L. secalinus*, *Leymus secalinus*. The data are presented as mean ± standard error (SE) of three independent experiments. ** *p* ≤ 0.01; *** *p* ≤ 0.001; NS, no significance. Different lowercase letters mean significant difference (*p* ≤ 0.05) between same species under different grazing intensities. Different uppercase letters mean significant difference (*p* ≤ 0.05) between different species or different grazing intensities.

**Figure 4:**
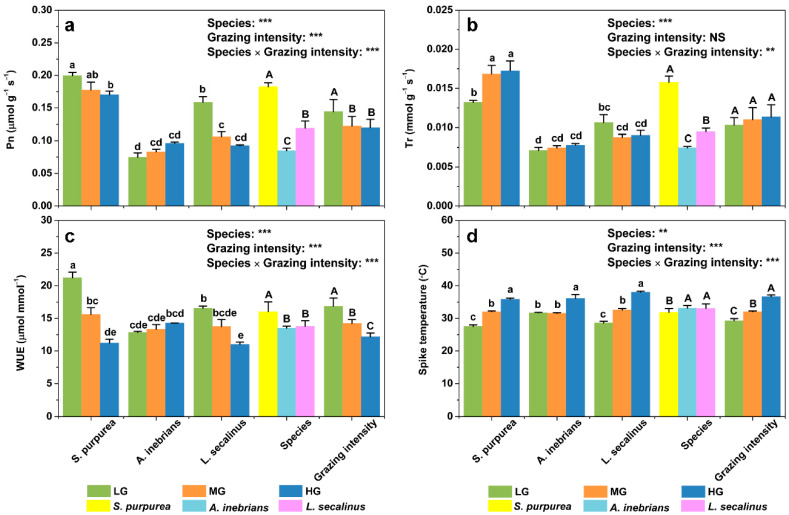
Mean values of photosynthesis parameters including the net photosynthetic rate (Pn) (**a**), transpiration rate (Tr) (**b**), water use efficiency (WUE) (**c**), and spike temperature (T_s_) (**d**) of the spikes of *S. purpurea*, *A. inebrians* and *L. secalinus* in response to grazing intensity (LG, light grazing; MG, medium grazing; HG, heavy grazing). Note: *S. purpurea*, *Stipa purpurea*; *A. inebrians*, *Achnatherum inebrians*; *L. secalinus*, *Leymus secalinus*. The data are presented as mean ± standard error (SE) of three independent experiments. ** *p* ≤ 0.01; *** *p* ≤ 0.001; NS, no significance. Different lowercase letters mean significant difference (*p* ≤ 0.05) between same species under different grazing intensities. Different uppercase letters mean significant difference (*p* ≤ 0.05) between different species or different grazing intensities.

The ANOVA results showed that the effects of species on the Pn were highly significant (*p* ≤ 0.001) in both the leaves and spikes (Figure 3a and Figure 4a). Furthermore, grazing intensity significantly affected spike Pn (*p* ≤ 0.001), whereas no significant differences were found in the leaf Pn (*p* > 0.05). The daily mean value of the leaf Pn of *L. secalinus* was the greatest under heavy grazing (HG), was significantly lower under medium grazing (MG) and was much lower under light grazing (LG). There were similar trends in the daily mean value of the leaf Pn of *S. purpurea*. In contrast, the daily mean value of the leaf Pn of *A. inebrians* was significantly greater under LG than under MG and HG. Interestingly, the daily mean value of the spike Pn of *S. purpurea* under HG was not significantly different from that under MG but lower than that under LG. Similarly, compared with those under LG, the daily mean values of the spike Pn of *L. secalinus* were significantly lower under MG and HG. Conversely, there were no significant differences in the daily mean value of the spike Pn of *A. inebrians* under LG, MG or HG. Compared with those under LG, the mean values of the spike Pn were significantly lower under MG and HG. However, there were no significant differences in the mean values of the leaf Pn under LG, MG and HG. Furthermore, compared with the mean values of the Pn of *S. purpurea*, the mean values of the Pn of *A. inebrians* and *L. secalinus* were 22.21% and 39.12% lower (*p* ≤ 0.001) in the leaves, respectively, and were 53.52% and 34.62% lower (*p* ≤ 0.001) in the spikes, respectively.

#### 2.2.2. Transpiration Rate

**Figure 5:**
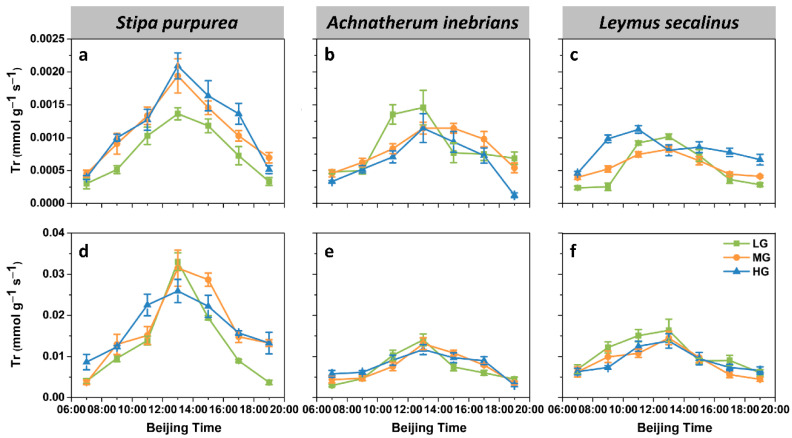
Diurnal dynamics of the transpiration rate (Tr) of the leaves and spikes of *S. purpurea* (**a**,**d**), *A. inebrians* (**b**,**e**) and *L. secalinus* (**c**,**f**) in response to grazing intensity (LG, light grazing; MG, medium grazing; HG, heavy grazing). The data are presented as mean ± standard error (SE) of three independent experiments.

The diurnal variation of the leaf transpiration rate (Tr) was unimodal under each grazing intensity in all three species, peaking at 13:00, except for the leaf Tr under HG in *L. secalinus* (Figure 5a–c). The maximum leaf Tr in *L. secalinus* (0.0011 mmol g^−1^ s^−1^) was half of the maximum value in *S. purpurea* (0.0021 mmol g^−1^ s^−1^), while the corresponding value in *A. inebrians* was slightly more than half that in *S. purpurea* (0.0015 mmol g^−1^ s^−1^). The diurnal pattern of the spike Tr was the same as that of the leaf Tr (Figure 5d–f). The maximum value of the spike Tr occurred in *S. purpurea* (0.033 mmol g^−1^ s^−1^), which was slightly more than twice that of *A. inebrians* (maximum 0.014 mmol g^−1^ s^−1^) and *L. secalinus* (maximum 0.016 mmol g^−1^ s^−1^).

ANOVA revealed that the effects of species on the Tr were highly significant (*p* ≤ 0.001) in both the leaves and spikes (Figure 3b and Figure 4b). Additionally, grazing intensity significantly affected leaf Tr (*p* ≤ 0.01), whereas no significant differences were found in the spike Tr (*p* > 0.05). Compared with those under LG, the daily mean values of the leaf Tr of *S. purpurea* were significantly greater under MG and HG. There were similar trends in the daily mean values of the spike Tr of *S. purpurea*. The daily mean value of the leaf Tr of *L. secalinus* was significantly greater under HG than under LG and MG. However, the daily mean value of the leaf Tr of *A. inebrians* was lower under HG than LG and MG, but no significant differences were detected between MG and HG. Furthermore, there were no significant differences in the daily mean values of the spike Tr under LG, MG and HG, whether in *A. inebrians* or in *L. secalinus*. The mean values of the leaf Tr under MG and HG were significantly greater than those under LG, while no significant differences were detected in the mean values of the spike Tr under LG, MG and HG. Compared with the mean values of the Tr of *S. purpurea*, the mean values of the Tr of *A. inebrians* and *L. secalinus* were 24.89% and 37.55%% lower (*p* ≤ 0.001) in the leaves, respectively, and were 52.79% and 39.87% lower (*p* ≤ 0.001) in the spikes, respectively.

#### 2.2.3. Water Use Efficiency

**Figure 6:**
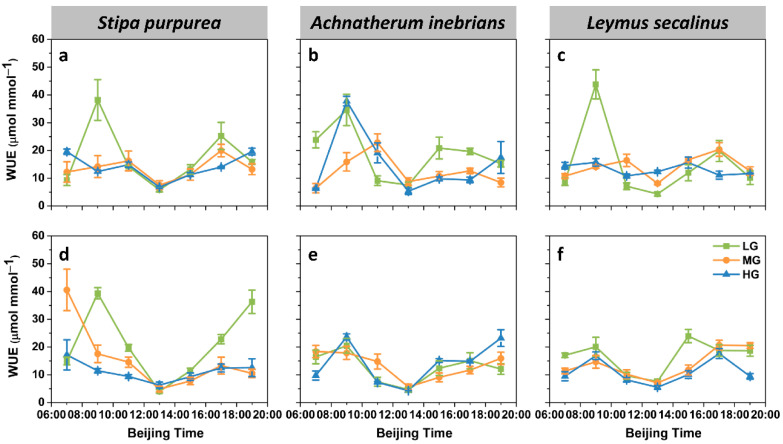
Diurnal dynamics of the water use efficiency (WUE) of the leaves and spikes of *S. purpurea* (**a**,**d**), *A. inebrians* (**b**,**e**) and *L. secalinus* (**c**,**f**) in response to grazing intensity (LG, light grazing; MG, medium grazing; HG, heavy grazing). The data are presented as mean ± standard error (SE) of three independent experiments.

The grazing intensity had significant effects on the diurnal patterns of the water use efficiency (WUE) (Figure 6). For the leaf WUE under LG and MG in the three species, the first peaks were observed at 09:00 and 11:00, but the second peaks were found at 15:00 and 17:00. However, the leaf WUE of *S. purpurea* under HG exhibited an inverse unimodal pattern. Conversely, the leaf WUE of *L. secalinus* under HG was relatively stable throughout the day. The leaf WUE of *A. inebrians* under HG peaked at 09:00 but exhibited a large reduction at 13:00 and then started to increase again at 19:00. Similarly, the spike WUE of both *S. purpurea* under LG and *A. inebrians* under HG exhibited the same trends. Moreover, the spike WUE of *S. purpurea* under MG and HG decreased from early morning (07:00) until noon (13:00) but then increased. There were similar trends in the spike WUE of *A. inebrians* under LG and MG. Unlike that which occurred for the spike WUE of the other two species, double peaks were observed for the spike WUE of *L. secalinus* at 09:00 and 15:00–17:00.

The ANOVA results showed that species significantly affected the spike WUE (*p* ≤ 0.001), whereas it was not significant for the leaf WUE (*p* > 0.05) (Figure 3c and Figure 4c). Moreover, the effects of grazing intensity were highly significant (*p* ≤ 0.001) in both the leaves and spikes. The minimum daily mean value of the leaf WUE was achieved under MG in *A. inebrians*; this value was 34.48% lower than the maximum daily mean value obtained under LG in the same species. However, no significant differences were detected in the daily mean values of the leaf WUE in either *S. purpurea* or *L. secalinus* under LG, MG and HG. Conversely, the daily mean value of the spike WUE of *S. purpurea* significantly decreased with increasing grazing intensity. A similar trend was observed in the daily mean values of the spike WUE of *L. secalinus*. The mean value of the spike WUE was the greatest under LG, was significantly lower under MG and was much lower under HG. There were similar trends in the mean values of the leaf WUE but none of the differences were significant between MG and HG. Furthermore, the mean value of the spike WUE was significantly greater in *S. purpurea* than that in *A. inebrians* and *L. secalinus.* However, there were no significant differences in the mean values of the leaf WUE among the three species.

#### 2.2.4. Organ Temperature

**Figure 7:**
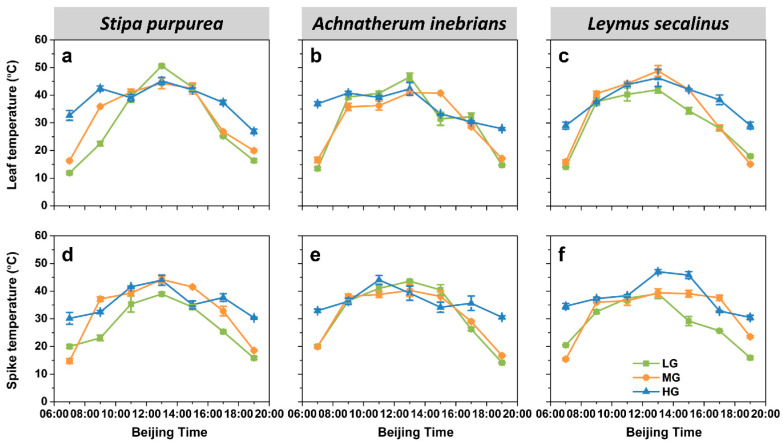
Diurnal dynamics of the leaf temperature (T_l_) and spike temperature (T_s_) of *S. purpurea* (**a**,**d**), *A. inebrians* (**b**,**e**) and *L. secalinus* (**c**,**f**) in response to grazing intensity (LG, light grazing; MG, medium grazing; HG, heavy grazing). The data are presented as mean ± standard error (SE) of three independent experiments.

The maximum values of both leaf temperature (T_l_) and spike temperature (T_s_) occurred at approximately 13:00 for all three species (Figure 7). Compared with T_l_ under HG ranged from 26.9 to 46.9 °C, T_l_ under LG and MG varied widely within the range of 11.5–51.3 °C. The trend of T_s_ was similar for each species, similar to the case for T_l_. T_s_ under LG and MG varied from 14.2 to 44.2 °C, while that under HG varied only from 30.2 to 47.0 °C.

ANOVA revealed that the effects of species and grazing intensity on T_l_ and T_s_ were highly significant (*p* ≤ 0.01, *p* ≤ 0.001) (Figure 3d and Figure 4d). The daily mean value of both T_l_ and T_s_ of *S. purpurea* was the lowest under LG, was significantly greater under MG and was much greater under HG. In addition, there were similar trends in both T_l_ and T_s_ of *L. secalinus*. In addition, the daily mean values of both T_l_ and T_s_ of *A. inebrians* were significantly greater under HG than those under MG but there were no significant differences in both T_l_ and T_s_ between MG and LG. The mean values of T_l_ and T_s_ significantly increased as grazing intensity increased. Additionally, the mean T_l_ in *A. inebrians* was not significantly different from that in *S. purpurea* but was significantly lower than that in *L. secalinus* (*p* ≤ 0.05). However, the mean T_s_ in *S. purpurea* was significantly lower (*p* ≤ 0.05) than that in *A. inebrians* and in *L. secalinus.*

#### 2.2.5. Correlations between Net Photosynthetic Rate, Transpiration Rate, Water Use Efficiency, and Organ Temperature under Grazing Intensity

**Figure 8:**
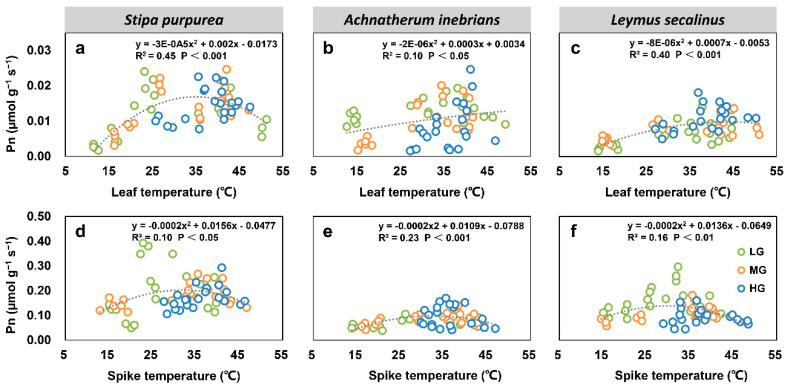
Correlations between leaf net photosynthetic rate (Pn) and leaf temperature (T_l_) (top), and between spike Pn and spike temperature (T_s_) (bottom) of *S. purpurea* (**a**,**d**), *A. inebrians* (**b**,**e**) and *L. secalinus* (**c**,**f**) under grazing intensity (LG, light grazing; MG, medium grazing; HG, heavy grazing).

**Figure 9:**
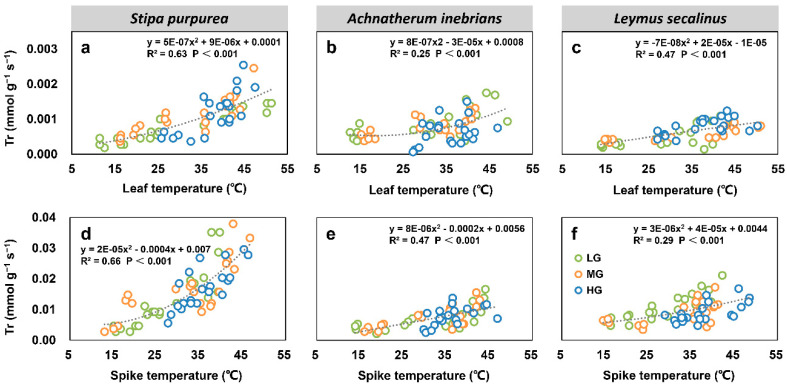
Correlations between leaf transpiration rate (Tr) and leaf temperature (T_l_) (top), and between spike Tr and spike temperature (T_s_) (bottom) of *S. purpurea* (**a**,**d**), *A. inebrians* (**b**,**e**) and *L. secalinus* (**c**,**f**) under grazing intensity (LG, light grazing; MG, medium grazing; HG, heavy grazing).

**Figure 10:**
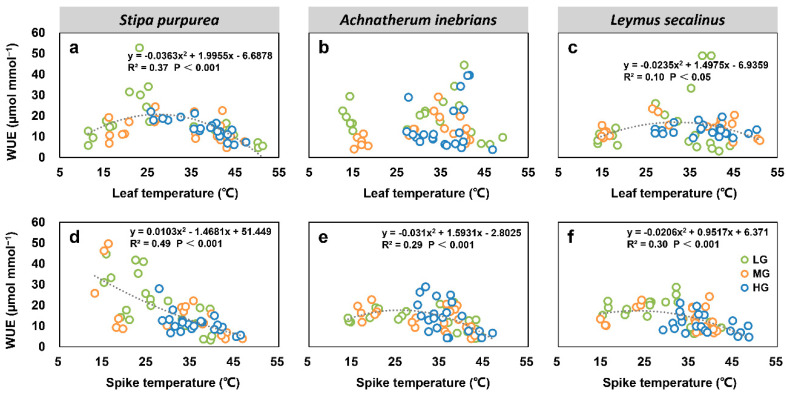
Correlations between leaf water use efficiency (WUE) and leaf temperature (T_l_) (top), and between spike WUE and spike temperature (T_s_) (bottom) of *S. purpurea* (**a**,**d**), *A. inebrians* (**b**,**e**) and *L. secalinus* (**c**,**f**) under grazing intensity (LG, light grazing; MG, medium grazing; HG, heavy grazing).

There were significant correlations between most leaf and spike photosynthesis parameters and the organ temperatures of *S. purpurea* (a, d), *A. inebrians* (b, e) and *L. secalinus* (c, f) under grazing intensity. The leaf Pn and spike Pn of three grass species under grazing intensity showed quadratic relationships with the leaf temperature and spike temperature (*p* < 0.05) (Figure 8). Similarly, the relationships between leaf and spike Tr and organ temperatures were significant for each species (*p* < 0.001) (Figure 9). Furthermore, leaf WUE and spike WUE of three grass species under grazing intensity also showed quadratic relationships with leaf temperature and spike temperature (*p* < 0.05); however, leaf WUE in *A. inebrians* had no correlation with the leaf temperature (Figure 10).

### 2.3. Chlorophyll Content

#### 2.3.1. Chlorophyll a, Chlorophyll b and Total Chlorophyll

ANOVA revealed that the effects of species and grazing intensity on the chlorophyll a (Chl *a*), chlorophyll b (Chl *b*), and total chlorophyll (TC) contents were significant (*p* ≤ 0.05 or *p* ≤ 0.001) in both the leaves and spikes (Table 1 and Table 2). Compared with those under HG, the Chl *a*, Chl *b* and TC contents in the leaves of *S. purpurea* were significantly lower under LG and MG. In contrast, the Chl *a* and TC contents in the leaves of *A. inebrians* were significantly lower under HG than those under LG and MG. Additionally, no significant differences were detected in the leaf Chl *b* content of *A. inebrians* among the three grazing intensities. Similarly, there were no significant differences in the leaf Chl *a* and Chl *b* contents under LG, MG and HG in *L. secalinus*. However, the TC content in the leaves of *L. secalinus* under MG was not significantly different from that under LG but was significantly lower than that under HG. Compared with that under LG, the mean Chl *a* content in the leaves under MG and HG was significantly lower. Furthermore, there were no significant differences in the mean Chl *b* and TC contents in the leaves under LG, MG and HG. On the other hand, the mean Chl *a*, Chl *b* and TC contents in the leaves were the greatest in *A. inebrians*, were significantly lower in *S. purpurea* and were much lower in *L. secalinus*.

The Chl *b* and TC contents in the spikes of *S. purpurea* under MG were not significantly different from those under HG but were significantly lower than those under LG (Table 2). Furthermore, the Chl *b* content in the spikes of *A. inebrians* significantly decreased with increasing grazing intensity. There were similar trends in the TC content in the spikes of *A. inebrians*, but none of the differences between LG and MG were significant. Moreover, there were no significant differences in the Chl *a* content in the spikes of either *S. purpurea* or *A. inebrians* among the three grazing intensities. The Chl *a*, Chl *b* and TC contents in the spikes of *L. secalinus* under MG were not significantly different from that under HG but lower than that under LG. In addition, compared with those under LG, the mean Chl *a*, Chl *b* and TC contents in the spikes were significantly lower under MG and HG. On the other hand, the mean Chl *a* and TC contents in the spikes were the greatest in *S. purpurea*, were significantly lower in *A. inebrians* and were much lower in *L. secalinus*. However, the mean Chl *b* content in *A. inebrians* was not significantly different from that in *S. purpurea* but greater than that in *L. secalinus*.

#### 2.3.2. Total Carotenoid Content, the chl a/chl b Ratio and TC/Total Carotenoid Ratio

The ANOVA results showed that the effects of species and grazing intensity on the total carotenoid (Car) content, chl *a*/chl *b* ratio (Chl *a*/*b*), and TC/Car ratio (TC/Car) were highly significant in both the leaves and spikes (*p* ≤ 0.05 or *p* ≤ 0.001), with the exception that they were not significant for the Car content and the Chl *a*/*b* in the spikes (Table 1 and Table 2). The Car content in the leaves of *S. purpurea* and *A. inebrians* significantly decreased as grazing intensity increased. There was a similar trend in the leaf Car content for *L. secalinus* but none of the differences were significant. Conversely, the Chl *a*/*b* in the leaves of *L. secalinus* significantly decreased with increasing grazing intensity, but no significant differences were detected in the leaf Chl *a*/*b* in either *S. purpurea* or *A. inebrians* under LG, MG and HG. In addition, the TC/Car in the leaves was markedly lower under LG and MG than under HG in both *S. purpurea* and *L. secalinus*, whereas grazing intensity had no effect on the TC/Car in *A. inebrians*. The mean Car content in the leaves significantly decreased as grazing intensity increased. There were similar trends in the mean Chl *a*/*b* in the leaves but none of the difference was significant between LG and MG. Conversely, the mean TC/Car in the leaves under MG was not significantly different from that under LG but lower than that under HG. Additionally, the mean Car content in the leaves was the greatest in *A. inebrians*, while that was the lowest in *L. secalinus*. In contrast, the mean TC/Car in the leaves was the lowest in *A. inebrians*, whereas that was the greatest in *L. secalinus*. Furthermore, the mean Chl *a*/*b* in the leaves of *A. inebrians* was not significantly different from that of *S. purpurea* but was lower than that of *L. secalinus*.

The Car content in the spikes of *S. purpurea* significantly increased with increasing grazing intensity (Table 2). Although the Car content in the spikes of *L. secalinus* followed the same trend, none of the differences were significant. Conversely, the Car content in the spikes of *A. inebrians* significantly decreased as grazing intensity increased. The Chl *a*/*b* in the spikes was the greatest under MG, was lower under HG and was much lower under LG in both *S. purpurea* and *L. secalinus*. However, the Chl *a*/*b* in the spikes of *A. inebrians* significantly increased with increasing grazing intensity. The TC/Car in the spikes under MG was not significantly different from that under HG but was significantly lower than that under LG in both *S. purpurea* and *L. secalinus*. However, the TC/Car in the spikes of *A. inebrians* significantly increased with increasing grazing intensity. Compared with those under LG, the mean Chl *a*/*b* in the spikes were greater under MG and HG. Conversely, the mean TC/Car in the spikes under MG and HG was lower than that under LG. In addition, there was no significant difference in the mean Car content in the spikes under LG, MG and HG. The mean Car content in the spikes of *S. purpurea* was significantly greater than that of *A. inebrians* and *L. secalinus*. Furthermore, the mean TC/Car in the spikes of *A. inebrians* was greater than that of *S. purpurea* and *L. secalinus*. However, no significant differences in the mean Chl *a*/*b* in the spikes were detected among any of the three species.

## 3. Discussion

### 3.1. Effect of Grazing Intensity on Photosynthesis Parameters

Photosynthesis is the most fundamental and intricate physiological process in all green plants [3]. Diurnal dynamic patterns in photosynthesis parameters can indicate the ability of plants to maintain internal balance while responding to changes in environmental conditions [30,31]. In this study, the dual-peak diurnal pattern of leaf and spike Pn on sunny days might be due to photoinhibition under high-PAR conditions [32]. The decrease in Pn after the first peak might have resulted from an increase in PAR that simultaneously escalated the Ta, T_l_ and T_s_ [33]. In addition, as a self-regulatory ecological adaptation of plants, the midday depression of Pn may be the result of photoinhibition; midday depression has often been attributed to relatively high irradiance, high temperature, and low RH [31,33]. The unimodal diurnal patterns of the Tr, PAR, and Ta suggest that plants may self-adjust and deploy a protective mechanism to avoid the damage caused by strong PAR and high temperature [34]. Furthermore, the trends of the T_l_ and T_s_ patterns were the same as those of the Tr patterns, demonstrating that each species reacted via stomatal opening (consequently increasing the Tr) to reduce the amount of damage to their leaves and spikes caused by high T_l_ and T_s_, which increased sharply because of high PAR.

With respect to the daily mean values of the leaf Pn, *S. purpurea* and *L. secalinus* presented consistent significant increases with grazing intensity (Figure 3a). The increase in the Pn at the leaf-level may be attributed to recovery from grazing-induced damage or from the increased production of energy for regrowth, namely, compensatory photosynthesis [35,36,37,38]. Compensatory photosynthesis is defined as an essential mechanism not only for plant leaf regrowth but also for plant tolerance to herbivores [8,35,36,39]. Hence, this increase resulted from a decrease in leaf area due to livestock feeding, which suggests that both species could promote the accumulation of assimilates, could improve grazing tolerance, and photosynthesized at a sufficient level to maintain the Pn under certain grazing intensities. Our results were consistent with those of other studies [8,10,38]. Interestingly, changes in physiological capacity of plant with increased grazing intensity can vary substantially among different rangeland ecosystems, and may be related to site- and species-specific features [40].

*A. inebrians*, commonly referred to as drunken horse grass, is a common toxic perennial grass species related to the narcosis of livestock that graze on grasslands in Northwest China [41,42]. Livestock that were fed or grazed on *A. inebrians* exhibited symptoms of intoxication [41,43]. The leaves of *A. inebrians* were typically older because grazing animals preferred *S. purpurea* and *L. secalinus* over *A. inebrians* when herbage was highly available. However, in the case of MG and HG, the leaf area of the old photosynthesizing leaves and the Pn decreased because of foraging or trampling by livestock. This offered a good explanation for why the daily mean values of the leaf Pn of *A. inebrians* decreased with increasing grazing intensity. Grazing-induced variation in plant composition will be critical for predicting the secondary production robustness estimates in semi-arid grassland area [44].

Transpiration plays a key role in the energy and water balance of land surface [45]. With increasing grazing intensity, the increases in the daily mean values of the leaf Tr of *S. purpurea* and *L. secalinus* was associated with the increase in the daily mean values of the T_l_. As a passive outcome of energy balance over plant body parts, T_l_ directly influences plant metabolic activities and energy management [46,47]. However, a T_l_ exceeding the optimum inhibits the enzymatic activity and chemical metabolism of cells, thus affecting gas exchange and photosynthesis in plants [48,49]. In this study, grazing can reduce grassland vegetation coverage and thus increasing the Ta and solar radiation in plant microhabitats, consequently leading to an increase in T_l_ [50,51]. Additionally, previous studies demonstrated that transpiration can substantially lower the T_l_ via evaporation of water from the leaf surface [52]. According to Urban et al. (2017) [53], evaporative cooling of transpiring leaves led to reductions in T_l_ of up to 9 °C in well-watered poplar but only 1 °C in drought-stressed poplar and loblolly pine. There was no significant difference in the daily mean values of leaf WUE of both *S. purpurea* and *L. secalinus*, showing that there were sufficient available water resources for plants in the environment. Therefore, the increase in the leaf Tr could effectively lower the corresponding T_l_ in an attempt to avoid heat damage attributed to high temperature. It has also been documented that both the Pn and Tr increased in the absence of water stress [54]. That is the reason why the leaf Pn and Tr of *S. purpurea* and *L. secalinus* increased with increasing grazing intensity.

Unlike that which occurred for *S. purpurea* and *L. secalinus*, the daily mean values of the leaf Pn and leaf Tr of *A. inebrians* significantly decreased as grazing intensity increased, and the leaf WUE followed the same trend for the same species, indicating that *A. inebrians* was subjected to water stress caused by grazing intensity. However, the T_l_ of *A. inebrians* increased with increasing grazing intensity. Our findings were in agreement with the results of prior studies showing that water stress reduced the Pn and Tr; in turn, the reduced Tr increased the T_l_ [54,55,56].

In addition to foraging, livestock trampling can directly impose strong mechanical stress on plants, resulting in physical damage to the leaves, shoots, stems, and roots [57,58,59]. Compared with the leaves, the spikes for each species were located at the top of the reproductive branches of the stems. In the absence of trampling, owing to their distinct spatial location, spikes are favored for the interception and capture of light and CO_2_. However, deer injure stems and roots by trampling them, causing abrasion and bending under light trampling and distortion, chlorosis, stunting, and even breakage and rupture under heavy trampling, consequently preventing the plants from transporting water and nutrients from the roots to the spikes [6,57,60]. Hence, the greater the grazing intensity was, the more severe the damage to the stems. Additionally, studies have showed that the trampled plants produced new leaves in a short time following trampling [57,58]. Hence, a portion of the water and nutrients transported from the roots should also be supplied to new lower-canopy leaves for compensatory growth, which in turn attracts livestock to forage once again and thus resulting in repeated trampling on vegetation. Aside from including chloroplasts necessary for photosynthesis in any plant, the photosynthetic tissues of spike also include stomata through which CO_2_ is be taken up by plants [3,61]. However, stomata are present only in the green parts of spikes and are distributed mainly in the glumes, awns, and lemmata [15,19,62]. Trampling-induced damage to the integrity of chloroplast structure and function reduces the spike photosynthetic area as well as spike photosynthesis [6]. Therefore, the daily mean values of the spike Pn of *S. purpurea* and *L. secalinus* significantly decreased with increasing grazing intensity. Owing to its toxicity, *A. inebrians* was typically not foraged by deer, thus avoiding or reducing the amount of injury caused by trampling, and accordingly, there was no significant difference in spike Pn.

Similar to that which occurred for the T_l_, the T_s_ for each species markedly increased with increasing grazing intensity as a result of the reduction in grassland vegetation coverage caused by grazing. As mentioned above with respect to leaf Tr, the increase in the daily mean values of the spike Tr in *S. purpurea* could lower the T_s_ to reduce the impact of heat injury on the Pn. However, there was no significant difference in the daily mean values of the spike Tr in *L. secalinus*. The reason may be associated with differences in tiller type of the two graminoids. *S. purpurea* is a dense clump grass with tiller nodes located on the soil surface, resulting in the formation of dense clumps. However, as a typical perennial rhizomatous grass species, *L. secalinus* has a guerrilla growth form and is one of the plants from long rhizomes with the strongest propagation ability [63,64]. *L. secalinus* typically has only one main rhizome that is very deep, located below 15–32 cm underground [65,66]. Therefore, water absorbed by the main rhizome of *L. secalinus* from deep underground supplements the increased water consumption due to the increase in the spike Tr, helping to lower the T_s_ and thus maintaining normal physiological activity.

Deer mainly foraged and trampled the palatable *S. purpurea* and *L. secalinus* following grazing, eliminating the competition between *A. inebrians* and the other two species, which somewhat reduced damage to the vegetation coverage in *A. inebrians* due to trampling. Only under HG did vegetation coverage of *A. inebrians* decrease significantly. This could be confirmed by the daily mean value of the T_s_ of *A. inebrians* under HG being markedly greater than that under LG and MG, while no significant difference was detected between LG and MG. This may explain why there was no significant difference in the spike Tr in *A. inebrians*.

Our results showed that the daily mean values of the spike WUE of both *S. purpurea* and *L. secalinus* significantly decreased with increasing grazing intensity. In addition, reductions in the WUE were related to changes in the Pn and Tr because it was affected by the coupling of photosynthesis and transpiration, which was regulated by the stomata [67,68]. On the other hand, reductions in the WUE indicated a decrease in the amount of biomass produced per unit volume of water consumed, as well as a reduction in the efficiency of water resource use by plants [69,70]. There was no significant difference in either the spike Pn or spike Tr of *A. inebrians*, leading to no significant differences in spike WUE.

With respect to species differences, ANOVA revealed that leaf and spike Pn of *S. purpurea* was the greatest among the three species, suggesting that *S. purpurea* performed the best in terms of synthesizing organic compounds (Figure 3a and Figure 4a). Similarly, leaf and spike Tr of *S. purpurea* was greater than that of the other two species, which means that *S. purpurea* consumed the greatest amount of water. However, a high Tr can reduce the increase in T_l_ and T_s_ at relatively high atmospheric temperatures in the environment, thus maintaining the optimal T_l_ and T_s_ to accelerate the production of photosynthates. No significant difference in leaf WUE was detected between *S. purpurea* and the other two species, indicating that the biomass produced per unit of water consumed for *S. purpurea* was not lower than that for *A. inebrians* and *L. secalinus*. High water consumption did not reduce the efficiency of the use of water resources in the environment by the leaves. However, the spike WUE of *S. purpurea* was significantly greater than that of the other two species, showing that *S. purpurea* assimilated the greatest amount CO_2_ while consuming the same amount of water, thus using water more efficiently. There was no significant difference in the T_l_ between *S. purpurea* and the other two species, but the T_s_ in *S. purpurea* was significantly lower than that in the other two species. It was concluded that under the same grazing intensity, *S. purpurea* could maintain a lower photosynthetic organ temperature and have the same or even stronger ability to regulate the temperature of its photosynthetic organs, which is important for maintaining the normal physiological function of photosynthetic organs.

### 3.2. Effect of Grazing Intensity on Photosynthetic Pigments

As the main photosynthetic pigment in chloroplasts as well as an essential biochemical component, chlorophyll is intimately involved in light harvesting, energy transfer and light energy conversion in photosynthesis [3,71]. Changes in chlorophyll content have critical consequences for estimating nutrient content in grasslands and understanding livestock feeding patterns and distribution [72,73]. There are two major varieties of chlorophyll, Chl *a* and Chl *b*, and the contents of them are vital indicators of plant photosynthetic capacity and health status [71,74]. There is considerable evidence indicating that plants exposed to adverse conditions present reduced contents of important photosynthetic pigments, particularly chlorophyll [54,75]. The stress-induced alterations in leaf chlorophyll content can be attributed to either impairment of pigment biosynthesis pathways or acceleration of pigment degradation [76,77,78]. The Chl *a*, Chl *b* and TC contents in the leaves of *S. purpurea* were significantly higher under HG than those under LG and MG, suggesting that the leaves presented improved photosynthetic efficiency by increasing the amount of photosystem components [79,80]. In addition, the increase in the chlorophyll content under HG could increase the photosynthetic potential to prepare the physiology for normal growth, which may be a physiological adaptation of this species to relatively severe grazing stress. Both the Chl *a* and Chl *b* contents in the leaves of *L. secalinus* did not differ significantly with increasing grazing intensity, indicating that they were not sensitive to grazing disturbance. However, the TC content in the leaves of *L. secalinus* under HG was significantly greater than that under MG, which suggests that grazing stimulated TC synthesis. Additionally, a marked reduction in the Chl *a* and TC contents in the leaves of *A. inebrians* could be a result of either inhibition of chlorophyll biosynthesis or acceleration of chlorophyll degradation [3,81]. This is in agreement with the results of a previous study [6].

The acclimation of the photosynthetic apparatus in higher plants is vital for optimizing the photosynthetic system under continuously changing environmental conditions, manifested by altering the structure, function, composition of photosystem II and its antenna size and by regulating the photosystem II: photosystem I ratio [82,83,84,85]. Thus, the Chl *a*/*b* related to antenna size could be an indicator of the degree of sun/shade acclimation and the structure of the photosynthetic apparatus, and its response to various stressors emphasizes the vital role of antenna size regulation in plant acclimation [79,84]. There was noticeable insignificance in the leaf Chl *a*/*b* of both *S. purpurea* and *A. inebrians*. This means that regulation of antenna size was not necessary, likely because of their acclimation to grazing stress. However, the decrease in the leaf Chl *a*/*b* of *L. secalinus* suggests that the antennas size of the photosystems was relatively large to make optimal use of the low internal light intensity [84,86].

Apart from participating in light harvesting, Car, as a quencher of chlorophyll triplets, singlet oxygen and reactive oxygen species, can protect the photosynthetic system against damage caused by stress, thus playing an essential photoprotective role [79,87]. The reduction in the Car contents in the leaves of both *S. purpurea* and *A. inebrians* was a response of grazing-induced stress that may lead to impairments of either photosynthetic activity or the protection mechanism for the photosynthetic system. However, the Car contents in the leaves of *L. secalinus* remained unchanged means that it was not impaired by grazing stress [81]. TC/Car, which is a good indicator of plant stress detection and tolerance, is sensitive to photooxidative damage [88,89]. A pronounced increase in the leaf TC/Car in both *S. purpurea* and *L. secalinus* was evident with an increase in grazing intensity, while no significance was detected in *A. inebrians*, indicating that *S. purpurea* and *L. secalinus* experience a lower intensity of oxidative stress than *A. inebrians* does with increasing environmental stress [90].

Low temperature and high UV-B radiation in high-mountain habitats induce photoinhibition [91]. The increase in PAR is the result of the decrease in the vegetation coverage caused by grazing. Moreover, compared with leaves, spikes contribute more to light interception because of their distinct spatial position. Thus, excessive absorbed light energy usually results in more photoinhibition of chloroplasts in the spikes than in the leaves [91,92]. Previous studies have shown that both low temperature and high UV-B radiation led to a decrease in the levels of Chl *a* and Chl *b* through chloroplast damage or disrupt chlorophyll biosynthesis [93,94]. Furthermore, Chl *b* is more sensitive to low temperature and high UV-B radiation than Chl *a* is [95,96]. This may explain why no significant variation was detected in the Chl *a* content in the spikes of either *S. purpurea* or *A. inebrians* in our study, while the Chl *b* contents in the spikes significantly decreased with increasing grazing intensity, thereby resulting in a decrease in the TC contents in the spikes of both species. Chl *a* and Chl *b* contents indicate a plant’s photosynthetic capability and health [74]. Photoinhibition can be divided into two processes: photodamage and the repair process [97]. The Chl *a*, Chl *b*, and TC contents in the spikes of *L. secalinus* were lower under MG than LG, suggesting that this decrease may be due to the impairment in spike chloroplasts induced by low temperature and increased UV-B exposure following grazing. However, these indicators under MG were lower than those under HG. This means that *L. secalinus* has the ability to restore its chlorophyll content rapidly, which not only could help maintain a certain amount of photosynthetic pigment but also could weaken the effects of grazing on spike photosynthesis.

Chl *b* is the main component of LHCII, acting as an antenna complex by transferring light energy to the reaction center of PSII [98]. In this study, an increased Chl *a*/*b* in the spikes of the three species is indicative of the preferential degradation of LHCII, which is an essential photoprotective mechanism for the photosystems; this degradation results in an increased proportion of excitation energy transferred to the PSII reaction center and thus decreasing absorption of light energy for ultimately protecting the photosystems and enabling plants to survive under adverse conditions [99,100,101]. In addition, this indicates a decrease in the size of the antenna complex associated with PSII, which reduces PSII excitation within the antenna system and thus preventing the dissipation of wasteful energy via photoprotective mechanisms that are used to relieve PSII excitation [101,102].

As noted above, Cars play an essential role not only in the photosynthesis process but also in the protection of thylakoid membranes against damage to the photosynthetic system induced by excess solar energy absorbed by photosynthetic pigments. The increase in the Car content in the spikes of *S. purpurea* indicates excitation energy transfer from chlorophyll molecules to Car molecules (i.e., chlorophyll zeaxanthin heterodimers), thus ensuring safe dissipation of excess energy [103,104]. As mentioned above with respect to leaf Cars, the decreased Car content in the spikes of *A. inebrians* suggests impairment in both photosynthetic activity and protective mechanisms, while that of *L. secalinus* was not sensitive to grazing stress. Given that the TC/Car can be considered a good indicator for evaluating environmental stress in plants and for evaluating the photosynthetic apparatus, the decreased TC/Car in the spikes of both *S. purpurea* and *L. secalinus* indicates an increase in photoprotection as well as protection of the photosynthesis apparatus via improved thermal dissipation of excitation energy, thus increasing antioxidant protection under stress conditions [89,105,106]. However, the increased TC/Car in the spikes of *A. inebrians* means that oxidative stress increased as grazing intensity increased. Therefore, the increased Chl *a*/*b* as well as the decreased TC/Car suggests a better acclimation of *S. purpurea* and *L. secalinus* to increasing grazing intensity compared with that of *A. inebrians* [107]. In addition, shifts in dominant plant communities under moderate grazing intensity may benefit livestock production [108].

## 4. Materials and Methods

### 4.1. Study area and Experimental Design

**Figure 11:**
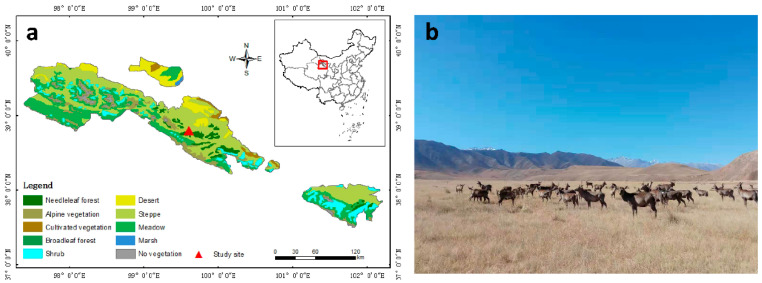
Map of study site. (**a**) Location of study site in Sunan County, the middle part in northern slope of the Qilian Mountains; (**b**) The grazing land and Gansu wapiti (*Cervus elaphus kansuensis*).

The study was carried out at the Sunan Deer Farm (SDF) (Figure 11a; 38°48′ N, 99°36′ E), which was established in 1958 and is located in Sunan County in Gansu Province, Northwest China. It is the first wild Gansu wapiti (*Cervus elaphus kansuensis*, hereinafter referred to as deer, Figure 11b) domesticating station, contains the largest artificial breeding group of deer and is one of the largest deer farms under the main management model of grazing in China [109]. The SDF is situated in the middle part in northern slopes of the Qilian Mountains, ranging from 2600 to 3000 m above sea level, and the rangeland was classified by Wu et al. as an “alpine typical steppe” [110]. The study area is characterized as having a plateau-mountain semiarid climate. The mean annual temperature is 3.6 °C, with a mean monthly temperature ranging from −10.4°C (in January) to 15.9 °C (in July), and there are approximately 127 frost-free days y^−1^ [28,111]. The mean annual precipitation is 260 mm, of which more than 86.4% falls during May–September. The annual pan evaporation rate in the study area is 1785 mm y^−1^.

The study area was located in a winter grazing pasture, which is open and flat, with an overall slope of < 3° occupying an area of approximately 160 hm^2^. The main feeding routes of deer were confirmed by a combination of observing the deer grazing behavior and inquiring the deer herders. Moreover, grazing intensity was selected according to the conditions of the vegetation and soil. Ten herds of deer aged 5–6 y with similar body sizes were observed. Livestock herds generally grazed during the daytime (from 6:30–7:00 to 19:30–20:00) and grazed for 12.5–13.5 h daily during the summer; in addition, the herds had a basically fixed activity time and feeding route and were kept in pens overnight. Land close to pens tends to be grazed more often than that far away from the pens. Therefore, the exit and entrance areas of the pasture were subject to the heaviest grazing intensity, and the grazing intensity decreased with increasing distance away from those areas. Thus, the sample plots with the grazing intensity treatments that included 1.45 AUM ha^−1^ (light grazing, (LG)), 3.45 AUM ha^−1^ (medium grazing, (MG)), and 6.90 AUM ha^−1^ (heavy grazing, (HG)), respectively, were established at 1250, 650 and 50 m away from the entrance of the pasture. There were three replicates per treatment, and each plot (100 × 100 m) covered an area of 1 hm^2^. *A. inebrians* (Hance) Keng was absolutely dominant within the upper layer grasses in the HG plot, the dominant species within the lower layer was *Convolvulus ammanii*, and the main associated species was *Cares* spp. The upper-layer grasses in the MG plot were dominated by *L. secalinus*, *A. inebrians* (Hance) Keng, *S. purpurea*, and *Agropyron cristatum* (L.) Gaertn. Furthermore, *A. inebrians* (Hance) Keng and *S. purpurea* were also dominant within the upper layer in the LG plot. The dominant species within the lower layer was *Artemisia frigida* in both the MG and LG plots. The soil type was mountain chestnut soil. The SDF is the experimental base of Lanzhou University. The animal management were in accordance with the rules and regulations of experimental field management protocols (file No: 2010-1 and 2010-2), which were approved by Lanzhou University.

### 4.2. Photosynthesis Parameters and Photosynthetic Pigments

At the center of the plot, we randomly threw the quadrat and then sampled where the quadrat fell. We randomly established three quadrats (1 × 1 m) within each plot. We randomly selected at least 3 individuals of similar plant height and age for each species in each quadrat and measured the environmental and photosynthesis parameters of the leaves and spikes via a portable photosynthesis system (CI-301PS; CID Inc., Vancouver, WA, USA). Measurements were made between 7:00 and 19:00 at 2 h intervals on cloud-free, sunny days during the last two weeks or so of July 1999. Healthy and mature third leaf and spikes at the top of the stems were retained in their natural position during the measurements. The photosynthesis parameters included the net photosynthetic rate (Pn), transpiration rate (Tr), water use efficiency (WUE), and organ temperature (leaf and spike temperature, T_l_ and T_s_); the WUE was derived as the ratio of the Pn to the Tr (Pn/Tr) according to the methods of Stępień and Kłobus [112]. Environmental parameters including the photosynthetically active radiation (PAR), air temperature (Ta), and air relative humidity (RH) were also measured. In addition, the photosynthetically active radiation sensor is a filtered GaAsP photodiode. The leaf chamber air temperature sensor is a Type K thermocouple. The air relative humidity sensor is a capacitive sensor along with a LM35DH temperature sensor.

The chlorophyll and carotenoid (Car) contents were determined after the leaves were ground in acetone (three samples, 1.5 g of leaf and spike fresh mass each per sample). The homogenate was centrifuged via a Nüve Füj 650 model centrifuge. The supernatant was separated, and the absorbances were measured via a Shimadzu UV-260 spectrophotometer to determine the chlorophyll a (Chl *a*), chlorophyll b (Chl *b*), total chlorophyll (TC), and Car contents, respectively. The Chl *a*, Chl *b*, and TC contents were calculated according to the methods of Inskeep and Bloom [113], and the Car content was calculated according to the methods of Lichtenthaler [114]. Chl *a*/Chl *b* ratio (Chl *a*/b) and TC/total Car ratio (TC/Car) were subsequently calculated.

### 4.3. Data and Statistical Analysis

The data were presented as mean ± standard error (SE). A two-way ANOVA was used to assess the effects of species, grazing intensity and their interaction on photosynthesis parameters and photosynthetic pigments of leaves and spikes. Regression analysis was used to establish relationships among the photosynthesis parameters. All statistical analyses were performed using SPSS 20.0 (SPSS, Inc., Chicago, IL, USA). *p*-values < 0.05 were considered to be statistically significant.

## 5. Conclusions

The findings from this study suggest that moderate grazing enhanced leaf photosynthetic capacity in *S. purpurea* and *L. secalinus* but reduced capacity in *A. inebrians*. However, spike photosynthetic capacity of three grass species decreased in response to grazing intensities. Grazing promoted leaf photosynthesis parameters of *S. purpurea* and *L. secalinus* as a result of compensatory photosynthesis. However, grazing reduced leaf photosynthesis parameters of *A. inebrians* due to its toxicity, which is a species strategy different from the other two species. Furthermore, spike photosynthesis parameters of three grass species decreased under grazing intensities owing to the distinct spatial location of spike. Among the three species, leaf and spike Pn and Tr of *S. purpurea* were the greatest, while organ temperature was the lowest, suggesting that *S. purpurea* performed the best in terms of synthesizing organic compounds and could maintain relatively low optimal organ temperatures because of increased water consumption, which is conducive to accelerating photosynthesis. This result indicated that *S. purpurea* displayed the greatest ability for leaf and spike photosynthetic capacity to withstand and acclimate to grazing disturbance. This study was unique in its exploration of grazing impact on spike photosynthesis parameters of grass species. More research on, for example, chlorophyll fluorescence, chloroplast ultrastructure, and antioxidant enzymes will therefore be necessary to clarify the differences in strategies between leaf and spike of different species in response to grazing disturbance.

## Figures and Tables

**Table 1:** Effects of different species and grazing intensities on leaf chlorophyll content.

Species	GI	Chl *a*	Chl *b*	TC	Car	Chl *a*/*b*	TC/Car
	(AUM ha^−1^)	(mg g^−1^ FW)	(mg g^−1^ FW)	(mg g^−1^ FW)	(mg g^−1^ FW)		
***S. purpurea***	LG	1.69 ± 0.09 c	0.67 ± 0.02 b	2.36 ± 0.08 c	0.42 ± 0.04 b	2.54 ± 0.19 abc	5.60 ± 0.28 c
	MG	1.72 ± 0.07 c	0.70 ± 0.06 b	2.41 ± 0.11 c	0.34 ± 0.03 bc	2.50 ± 0.20 abc	7.24 ± 0.51 c
	HG	2.05 ± 0.07 b	0.96 ± 0.10 a	3.01 ± 0.15 b	0.29 ± 0.03 c	2.17 ± 0.20 bc	10.40 ± 0.44 b
***A. inebrians***	LG	2.68 ± 0.14 a	0.96 ± 0.02 a	3.64 ± 0.15 a	0.58 ± 0.03 a	2.80 ± 0.12 abc	6.31 ± 0.25 c
	MG	2.43 ± 0.02 a	0.95 ± 0.01 a	3.38 ± 0.03 a	0.56 ± 0.01 a	2.56 ± 0.02 abc	6.04 ± 0.07 c
	HG	1.51 ± 0.08 c	0.77 ± 0.02 ab	2.29 ± 0.09 c	0.42 ± 0.01 b	1.96 ± 0.09 c	5.43 ± 0.09 c
***L. secalinus***	LG	0.65 ± 0.02 d	0.21 ± 0.02 c	0.85 ± 0.02 de	0.08 ± 0.00 d	3.22 ± 0.32 a	11.25 ± 0.32 b
	MG	0.45 ± 0.02 d	0.14 ± 0.01 c	0.59 ± 0.01 e	0.06 ± 0.00 d	3.15 ± 0.33 ab	9.74 ± 0.19 b
	HG	0.68 ± 0.00 d	0.32 ± 0.03 c	1.00 ± 0.04 d	0.05 ± 0.00 d	2.19 ± 0.22 b c	19.01 ± 1.18 a
**Mean**	*S. purpurea*	1.82 ± 0.07 B	0.78 ± 0.06 B	2.59 ± 0.12 B	0.35 ± 0.03 B	2.40 ± 0.11 B	7.75 ± 0.73 B
	*A. inebrians*	2.21 ± 0.18 A	0.89 ± 0.03 A	3.10 ± 0.21 A	0.52 ± 0.03 A	2.44 ± 0.13 B	5.93 ± 0.15 C
	*L. secalinus*	0.59 ± 0.04 C	0.22 ± 0.03 C	0.82 ± 0.06 C	0.06 ± 0.00 C	2.85 ± 0.22 A	13.33 ± 1.48 A
	LG	1.67 ± 0.30 A	0.61 ± 0.11 A	2.28 ± 0.41 A	0.36 ± 0.08 A	2.85 ± 0.15 A	7.72 ± 0.90 B
	MG	1.53 ± 0.29 B	0.60 ± 0.12 A	2.13 ± 0.41 A	0.32 ± 0.07 B	2.74 ± 0.15 A	7.67 ± 0.57 B
	HG	1.41 ± 0.20 B	0.68 ± 0.10 A	2.10 ± 0.30 A	0.26 ± 0.05 C	2.10 ± 0.10 B	11.61 ± 2.02 A
**Significance** **level**	Species	***	***	***	***	*	***
	GI	***	*	*	***	***	***
	Species × GI	***	***	***	*	NS	***

Note: The data are presented as mean ± standard error (SE) of three independent experiments. * *p* ≤ 0.05; ** *p* ≤ 0.01; *** *p* ≤ 0.001; NS, no significance. Abbreviations: *S. purpurea*, *Stipa purpurea*; *A. inebrians*, *Achnatherum inebrians*; *L. secalinus*, *Leymus secalinus.* GI, Grazing intensity; LG, light grazing; MG, medium grazing; HG, heavy grazing; Chl *a*, chlorophyll a; Chl *b*, chlorophyll b; TC, total chlorophyll; Car, total carotenoid; Chl *a*/*b*, chl *a*/chl *b* ratio; TC/Car, TC/Car ratio. Different lowercase letters mean significant difference (*p* ≤ 0.05) between same species under different grazing intensities. Different uppercase letters mean significant difference (*p* ≤ 0.05) between different species or different grazing intensities.

**Table 2:** Effects of different species and grazing intensities on spike chlorophyll content.

Species	GI	Chl *a*	Chl *b*	TC	Car	Chl *a*/*b*	TC/Car
	(AUM ha^−1^)	(mg g^−1^ FW)	(mg g^−1^ FW)	(mg g^−1^ FW)	(mg g^−1^ FW)		
***S. purpurea***	LG	0.55 ± 0.02 a	0.32 ± 0.03 a	0.87 ± 0.05 a	0.073 ± 0.007 b c	1.74 ± 0.13 ef	12.05 ± 0.92 ab
	MG	0.45 ± 0.01 ab	0.12 ± 0.00 cde	0.57 ± 0.01 bc	0.093 ± 0.009 b	3.65 ± 0.10 ab	6.22 ± 0.54 c d
	HG	0.48 ± 0.02 a	0.17 ± 0.01 bc	0.65 ± 0.02 b	0.143 ± 0.008 a	2.82 ± 0.14 cd	4.54 ± 0.15 d
***A. inebrians***	LG	0.35 ± 0.02 bc	0.32 ± 0.02 a	0.67 ± 0.04 b	0.090 ± 0.005 b	1.08 ± 0.08 f	7.47 ± 0.21 c
	MG	0.45 ± 0.01 ab	0.21 ± 0.01 b	0.65 ± 0.02 b	0.061 ± 0.003 cd	2.17 ± 0.12 de	10.73 ± 0.43 b
	HG	0.37 ± 0.04 b	0.09 ± 0.01 de	0.46 ± 0.04 cd	0.033 ± 0.002 e	3.92 ± 0.08 a	13.91 ± 1.09 a
***L. secalinus***	LG	0.35 ± 0.04 bc	0.15 ± 0.01 bcd	0.50 ± 0.05 cd	0.048 ± 0.005 de	2.28 ± 0.24 de	10.42 ± 0.75 b
	MG	0.20 ± 0.02 d	0.07 ± 0.00 e	0.27 ± 0.02 e	0.057 ± 0.001 cd	3.10 ± 0.33 bc	4.77 ± 0.41 cd
	HG	0.26 ± 0.02 cd	0.10 ± 0.00 de	0.36 ± 0.02 de	0.061 ± 0.003 cd	2.64 ± 0.17 cd	5.84 ± 0.06 cd
**Mean**	*S. purpurea*	0.49 ± 0.02 A	0.20 ± 0.03 A	0.70 ± 0.05 A	0.103 ± 0.011 A	2.74 ± 0.28 A	7.60 ± 1.18 B
	*A. inebrians*	0.39 ± 0.02 B	0.21 ± 0.03 A	0.59 ± 0.04 B	0.061 ± 0.008 B	2.39 ± 0.42 A	10.70 ± 0.99 A
	*L. secalinus*	0.27 ± 0.03 C	0.11 ± 0.01 B	0.37 ± 0.04 C	0.055 ± 0.003 B	2.67 ± 0.17 A	7.01 ± 0.90 B
	LG	0.41 ± 0.04 A	0.27 ± 0.03 A	0.68 ± 0.06 A	0.070 ± 0.007 A	1.70 ± 0.19 B	9.98 ± 0.75 A
	MG	0.37 ± 0.04 B	0.13 ± 0.02 B	0.50 ± 0.06 B	0.070 ± 0.006 A	2.97 ± 0.24 A	7.24 ± 0.93 B
	HG	0.37 ± 0.03 B	0.12 ± 0.01 B	0.49 ± 0.05 B	0.079 ± 0.017 A	3.13 ± 0.21 A	8.10 ± 1.50 B
**Significance** **level**	Species	***	***	***	***	NS	***
	GI	*	***	***	NS	***	***
	Species × GI	***	***	***	***	***	***

Note: The data are presented as mean ± standard error (SE) of three independent experiments. * *p* ≤ 0.05; ** *p* ≤ 0.01; *** *p* ≤ 0.001; NS, no significance. Abbreviations: *S. purpurea*, *Stipa purpurea*; *A. inebrians*, *Achnatherum inebrians*; *L. secalinus*, *Leymus secalinus*. GI, Grazing intensity; LG, light grazing; MG, medium grazing; HG, heavy grazing; Chl *a*, chlorophyll a; Chl *b*, chlorophyll b; TC, total chlorophyll; Car, total carotenoid; Chl *a*/*b*, chl *a*/chl *b* ratio; TC/Car, TC/Car ratio. Different lowercase letters mean significant difference (*p* ≤ 0.05) between same species under different grazing intensities. Different uppercase letters mean significant difference (*p* ≤ 0.05) between different species or different grazing intensities.

## Data Availability

Relevant data applicable to this research are within the paper.
